# Adding team-based financial incentives to the Carrot Rewards physical activity app increases daily step count on a population scale: a 24-week matched case control study

**DOI:** 10.1186/s12966-020-01043-1

**Published:** 2020-11-19

**Authors:** Emma Pearson, Harry Prapavessis, Christopher Higgins, Robert Petrella, Lauren White, Marc Mitchell

**Affiliations:** 1 Faculty of Health Sciences, School of Kinesiology, Western University, Arts & Humanities Building, Room 3R12B, London, Ontario N6A 5B9 Canada; 2 Ivey Business School, Western University, London, ON Canada; 3 Schulich School of Medicine and Dentistry, Western University, London, ON Canada; 4 Faculty of Medicine, The University of British Columbia, Vancouver, BC Canada; 5 Carrot Insights Inc., Toronto, ON Canada

**Keywords:** Social relatedness, Physical activity, mHealth, Public health, Behavioural economics, Rewards

## Abstract

**Background:**

Mobile health applications (mHealth apps) targeting physical inactivity have increased in popularity yet are usually limited by low engagement. This study examined the impact of adding team-based incentives (Step Together Challenges, STCs) to an existing mHealth app (Carrot Rewards) that rewarded individual physical activity achievements.

**Methods:**

A 24-week quasi-experimental study (retrospective matched pairs design) was conducted in three Canadian provinces (pre-intervention: weeks 1–12; intervention: weeks 13–24). Participants who used Carrot Rewards and STCs (experimental group) were matched with those who used Carrot Rewards only (controls) on age, gender, province and baseline mean daily step count (±500 steps/d). Carrot Rewards users earned individual-level incentives (worth $0.04 CAD) each day they reached a personalized daily step goal. With a single partner, STC users could earn team incentives ($0.40 CAD) for collaboratively reaching individual daily step goals 10 times in seven days (e.g., Partner A completes four goals and Partner B completes six goals in a week).

**Results:**

The main analysis included 61,170 users (mean age = 32 yrs.; % female = 64). Controlling for pre-intervention mean daily step count, a significant difference in intervention mean daily step count favoured the experimental group (*p* < 0.0001; η_p_^2^ = 0.024). The estimated marginal mean group difference was 537 steps per day, or 3759 steps per week (about 40 walking min/wk). Linear regression suggested a dose-response relationship between the number of STCs completed (app engagement) and intervention mean daily step count (adjusted R^2^ = 0.699) with each new STC corresponding to approximately 200 more steps per day.

**Conclusion:**

Despite an explosion of physical activity app interest, low engagement leading to small or no effects remains an industry hallmark. In this paper, we found that adding modest team-based incentives to the Carrot Rewards app increased mean daily step count, and importantly, app engagement moderated this effect. Others should consider novel small-teams based approaches to boost engagement and effects.

**Supplementary Information:**

The online version contains supplementary material available at 10.1186/s12966-020-01043-1.

## Introduction

The health benefits of physical activity (PA) are irrefutable and yet, widespread inactivity persists [1, 2]. Interventions that increase PA on population-levels are needed to help tackle this important public health issue [3]. As smartphone ownership increases (approaching 90% in the US) [4], so has the number of mobile health applications (mHealth apps) in the major app stores (over 325,000; 30% are PA apps) [5]. Part of the appeal of mHealth apps is their potential to reach large populations at relatively low cost [6]. Their effectiveness, however, is often limited by low user engagement with 90% of mHealth apps being deleted within 30 days [7–9]. A still limited number of RCT studies suggest a dose-response relationship exists between engagement and effectiveness, with greater app use associated with larger PA improvements [10–12]. Conversely, waning engagement has been linked with declining PA [10, 11, 13–15]. Recent advances in behavioural science provide a new framework from which to develop practical solutions to address this notorious mHealth app engagement problem.

Behavioural economics, a branch of economics shaped by insights from psychology [16–18], has stimulated renewed interest in financial health incentive interventions, such as rewarding people to walk more [19]. This increasingly common intervention is grounded in a behavioural economics concept called “present bias” which describes how individuals tend to place disproportionate emphasis on the present “cost” of a health behaviour (e.g., time) while discounting the future “benefits” of that behaviour (e.g., increased health) [16]. Behavioural economics suggests that providing timely financial incentives for behaviours with benefits that are largely delayed (e.g., PA) may encourage individuals to choose to engage in those behaviours rather than put them off [16, 18, 20]. Individual-level financial incentives for PA (e.g., incentives for personal PA achievements) have shown promising results. A recent meta-analysis of RCTs concluded that financial incentives increased PA in the short-term by up to 4000 steps per day, with some evidence of long-term (six or more months) and sustained (after incentives withdrawn) effects [21]. A number of studies also suggest team-based incentives (e.g., incentives for group achievements) may be efficacious as well [22]. Compared to individual incentives, team incentives have yielded better gym attendance, more PA, and greater weight loss in RCT settings [23–25]. Interestingly, Patel et al. (2018) found that combining individual and team (i.e. combined) incentives was more efficacious than individual or team incentives alone [26].

The notion of “aligning the thoughts or behaviours of individuals in a group” is another pertinent behavioural economics concept called “herd behaviour” [27]. Herd behaviour describes how individuals are more likely to follow others in decision making instead of making independent decisions (e.g., “My friend is going for a walk, so I probably should too.”) [21]. The tendency for humans to want to behave in ways that are consistent with the people in their social networks may be leveraged in an mHealth context, for instance: (a) by providing feedback on peers’ progress, and/or (b) with team-based incentives. Recent evidence, though, suggests that adding a social component to mHealth interventions does not necessarily translate into positive effects [7, 11, 28, 29]. It appears that mHealth features designed to increase social connectivity among participants *with no prior relationship* do not work as well as those delivered among people with *existing relationships* (e.g., work colleagues challenge each other in an online walking challenge) [7, 26, 29, 30]. Babcock et al. (2015) compared anonymous partners to partners with an existing social connection and found PA incentives were not as effective in the anonymous group, highlighting the importance of leveraging pre-existing social connections in mHealth interventions [24, 26, 30].

Despite their popularity, very little is known about the effectiveness of commercial PA apps (or their design features) since few have undergone rigorous peer-reviewed evaluation [10, 31]. Among the 15 studies included in the recent Petersen et al. (2019) review of PA apps, for example, only five examined commercially available ones (e.g., Fitbit, ‘Zombie, Run!’) despite there being over roughly 100,000 in the major app stores [31, 32]. Among these five, little consideration was given to the role of engagement as an effect moderator despite suggestions that intervention exposure is imperative and that greater engagement usually yields larger effects [33]. The Carrot Rewards app was a top tier Canadian app (i.e. 1.3+ million downloads, 500,000+ monthly active users (MAUs) as of May 2019) that rewarded users with loyalty points redeemable for consumer goods (e.g., gas, movies) for walking more. It was developed in partnership with the Public Health Agency of Canada as part of its Multi-Sectoral Partnership Approach to Healthy Living and Chronic Disease Prevention [34]. One of the stated objectives of the initiative was to conduct rigorous evaluations of the app intervention, including the impact of new features [12, 35]. In March 2018, Carrot Rewards launched a new social feature called ‘Step Together Challenges’ (STCs) to complement their existing walking program (called ‘Steps’) where individualized daily step goal achievements were rewarded with very small incentives ($0.04 CAD per day). STCs allowed users to invite a friend from their existing social network to participate in a collaborative walking challenge for bonus incentives ($0.40 CAD per week).

To enhance our understanding of mHealth interventions, and acknowledging how difficult it can be to conduct RCTs in fast paced commercial digital environments, non-RCT alternatives (e.g., quasi-experimental designs) have been recommended [31, 36, 37]. Quasi-experimental evaluations of “top tier” commercial apps (i.e. the top 2% of apps reporting more than 500,000 MAUs) [5] may provide particularly valuable insight into mHealth app engagement, it’s role in promoting health behaviours, and how it can be improved on a population scale. The primary objective of this study, then, was to examine the impact of adding team incentives to the Carrot Rewards app on mean daily step count. An important secondary objective was to determine whether a dose-response relationship existed between app engagement (i.e. STCs completed) and mean daily step count.

## Methods

### Study design and sample

A 24-week retrospective pre-post matched pairs design was used to examine the effect of adding STCs to the Carrot Rewards ‘Steps’ walking program on mean daily step count. Participants were drawn from the existing Carrot Rewards user base which included Canadians 13 years of age or older living in the three provinces the app was launched (i.e. British Columbia (BC), Newfoundland and Labrador (NL), Ontario (ON)). All participants had to have opted-into the ‘Steps’ walking program to be included in the study. The experimental group included participants using the STC feature for the first time between March 19 and April 16, 2018 (the first month STC was available). Control participants were drawn from the cohort of current Carrot Rewards users who had enabled the ‘Steps’ walking program but had not engaged in a STC during the study period. Control participants were matched with existing experimental participants on age (±1 yr), gender, province and baseline step count (±500 steps/d, so individuals with similar PA levels would be compared). Only one control user was selected to match to experimental users if they met each of the four criteria; therefore, one control user could be matched with multiple experimental users who shared the same age, gender, province and baseline daily step count. Notably, 10% of the study population with the highest matching ratios (more than 1:18 and up to 1:250) were excluded to minimize the experimental-control imbalance (for more details see Additional file 1). Sensitivity analyses were conducted with experimental-control participants matched 1:1 only as well to check if the imbalance influenced results.

The pre-intervention period was defined as the 12 weeks preceding experimental users’ first STC (Study Weeks 1–12). The intervention period included the 12 weeks following the initiation of the first STC (Study Weeks 13–24). Participants were required to have valid pre-intervention and intervention study periods, consisting of a minimum of four weeks of daily step count data in each period—a valid week was operationally defined as a minimum of four days with step counts between 1000 and 40,000 inclusive, as previously done [38]. A study flow chart is provided (Additional file 2). Ethical approval for this study was provided by Western University’s Human Research Ethics Board (#111252).

### Individual and team incentives

Upon downloading the free, commercial Carrot Rewards app, and following a two-week baseline period, Carrot Rewards users earned individual-level incentives in the form of loyalty points (redeemable for consumer goods like movies or groceries) each day they reached a personalized daily step goal (worth $0.04 CAD/day). Given finite reward budgets and a large user base, and to maximize program scalability and sustainability, the smallest possible loyalty point increment was selected (i.e. the app could not offer *less* than 1 point/d = $0.04 CAD/d). Previous research has suggested that as part of a multicomponent intervention this incentive magnitude could stimulate PA [12]. In addition, several RCTs have demonstrated positive effects with PA incentives worth $0.09 to $0.75 USD per day [23, 39–41]. Goals were initially set using the two-week baseline median (e.g., if a user’s baseline daily step count median was 5441 steps, their first goal would be rounded to 5400). See Mitchell et al. (2018) for a more full description of the goal setting approach, including how goals were progressed [42]. While small, incentives were tied to objectively measured PA and were given nearly instantaneously with a push notification using smartphone technology (e.g., linking data from native smartphone accelerometer with loyalty program application programming interfaces (APIs)). Manual entry of daily step count was not possible (e.g., from a pedometer) nor were participants able to set their own step goal, in order to ensure incentives were earned for meaningful PA efforts. To boost app engagement and PA, the ‘Steps’ walking program evolved with refinement of older features, as well as the introduction of new ones. For example, the algorithm used to calculate each user’s daily step goal was updated to be more personalized and adaptive [42].

**Fig. 1:**
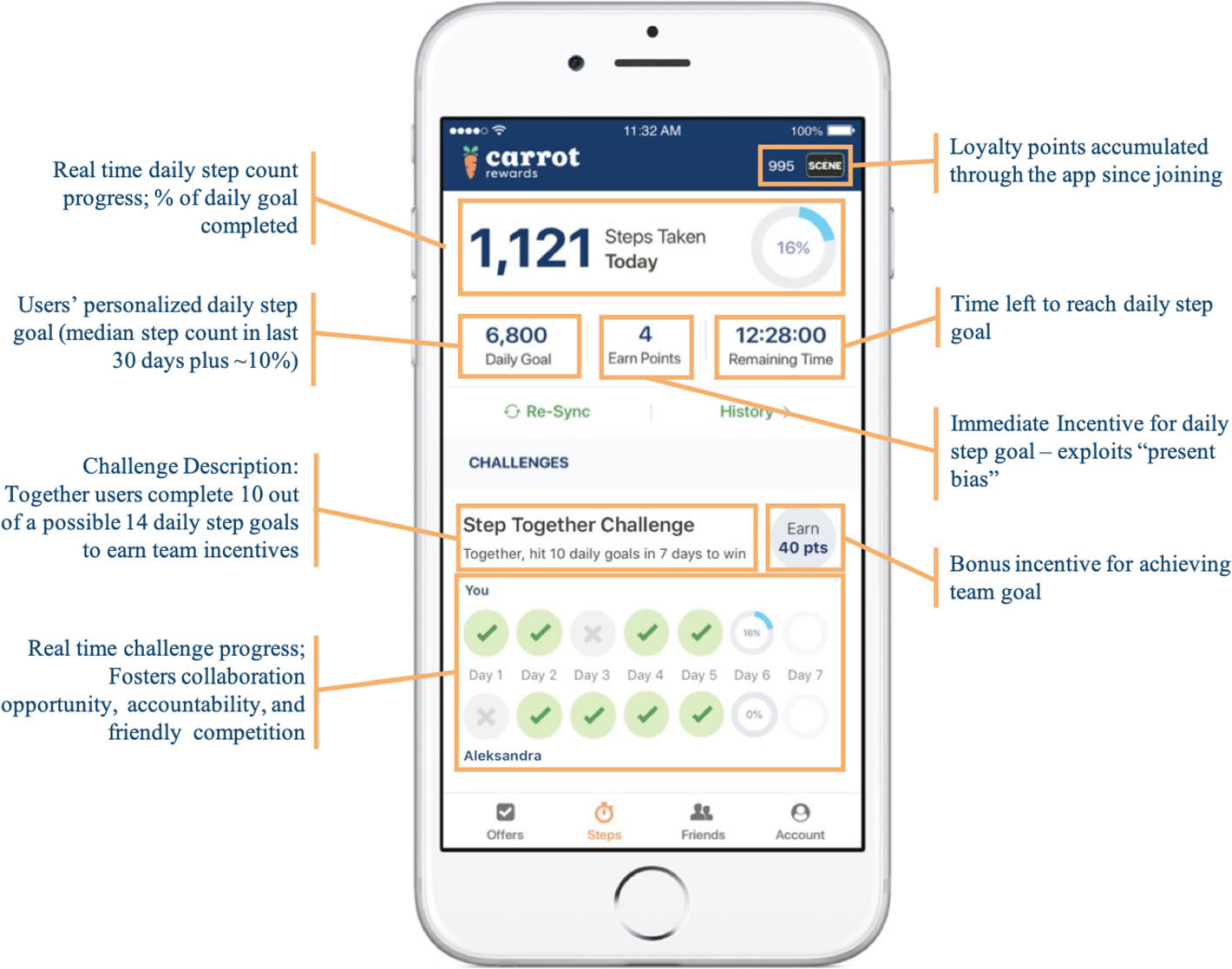
Carrot Rewards Step Together Challenge interface

STCs were introduced in March 2018 to allow users to collaboratively pursue team-based goals with a peer of their choosing for additional rewards (i.e. a pre-existing friend they had already connected with on the app). Users participating in a STC could each earn a bonus incentive worth $0.40 CAD for together reaching 10 individual daily step goals in a seven-day period (e.g., Partner A completes four goals and Partner B completes six goals in a week; Fig. 1). Users could only participate in one STC at a time. The app allowed users to see their partner’s daily step progress in real time, as well as their own, though users could not communicate about their shared progress in-app (this needed to be done through other means e.g., text messages, in-person, etc.). Over the course of 12-weeks, STC participants could earn a *maximum* of $9.76 CAD in points. In addition to promoting social support, the STC feature integrated other behaviour change techniques as well including goal setting/review, self-monitoring and demonstration. For more app design detail, completed Mobile App Rating Scale (MARS self-score 4.23/5; for understanding app quality, aesthetics and functional appeal) [43] and App Behavior Change Scale (ABACUS self-score 4.5/5; for measuring potential to change behaviour) [44] are provided (Additional files 3 and 4).

### Outcomes

The primary outcome was mean daily step count as measured by built-in smartphone accelerometers. In recent validation studies, the iPhone step counting feature, as well as those for Android smartphones were accurate in laboratory and field conditions [45–48]. Duncan et al. (2018) did determine, however, that steps were under-estimated by the iPhone step counting feature in their free-living condition by approximately 20%, or 1340 steps/day. According to the study authors this likely reflects not carrying the iPhone continually throughout the day rather than inaccuracy in the step counting feature; if adherence can be optimized, they suggest, then smartphones may be suitable for PA evaluations. Self-reported demographics (i.e. age, gender, province) and number of STCs completed were also collected. Number of STCs completed was defined as any STC that was started and finished within the seven-day window, irrespective of whether the Challenge was completed successfully or not. To finish the Challenge, a user simply needed to open the app to facilitate app vs. smartphone data synchronization.

### Statistical analyses

Chi-square and independent t-tests were conducted to examine group equivalency on demographic measures. Controlling for pre-intervention mean daily step count, ANCOVA was performed to examine group differences in intervention period mean daily step count. Data were expressed in estimated marginal means (95% CI). To complement the ANCOVA and increase internal validity (i.e. the extent to which causality can be established) in this quasi-experimental study a number of analysis phase strategies recommended by Handley et al. (2018) were deployed [49]. First, a pairwise t-test examined the mean daily step count change over time (pre-intervention vs. intervention) for each group. Second, ANCOVA and pairwise t-test sensitivity analyses were performed with (a) users with complete data sets only (highly-engaged users with valid step count data for all 24 study weeks), as well as (b) participants with a 1:1 control to experimental matching ratio only (vs. in the overall sample where controls were matched with up to 18 experimental users). Finally, linear regression was performed to determine whether a relationship existed between the number of STCs completed and intervention period mean daily step count. Statistical significance were two-sided and set at 0.05 [50]. Reported effect sizes followed Cohen’s (1988, 1992) criteria; Cohen’s *d*: small = 0.20, medium = 0.50, large = 0.80, Cramer’s V for chi squared: small = 0.10, medium = 0.30, large = 0.50, partial eta squared: small = 0.01, medium = 0.06, large = 0.14 [51, 52]. Statistical analyses were performed using IBM SPSS Statistics Version 25.

## Results

### Sample characteristics

Study sample characteristics, mean baseline daily step count, and mean number of valid weeks in the pre-intervention and intervention period are in Table 1. Group differences in age, gender and province were detected likely due to the large sample size, though effect sizes were very small. As well, the mean baseline daily step count for our study sample was higher than for Carrot Rewards users in general possibly because they were a more engaged sub-group. Lastly, experimental users were different than controls by virtue of the fact that they were early STC adopters (thus arguably ‘more engaged’). The comparable number of valid weeks in the pre-intervention period (11.16 and 10.81 for experimental and controls, respectively), on the other hand, suggest they might have been similarly engaged.

**Table 1:** Study sample (experimental vs. control) and overall Carrot Rewards user population characteristics

Category	Experimental (*n* = 39,355)	Control (*n* = 21,815)	Study Sample (*n* = 61,170)	Overall (*n* = 870,255)
*Age (mean ± SD)*^a^	32.13 ± 11.18	32.60 ± 11.20	32.3 ± 11.19	33.7 ± 11.6
13–17 years	1151 (2.9%)	621 (2.8%)	1772 (2.9%)	27,452 (4%)
18–24 years	9848 (25.0%)	5096 (23.4%)	14,944 (24.4%)	178,439 (24%)
25–34 years	15,102 (38.4%)	8278 (37.9%)	23,380 (38.2%)	241,746 (32%)
35–44 years	7332 (18.6%)	4374 (20.1%)	11,706 (19.1%)	140,785 (19%)
45–54 years	3854 (9.8%)	2267 (10.4%)	6121 (10%)	97,143 (13%)
55–64 years	1729 (4.4%)	957 (4.4%)	2677 (4.4%)	52,023 (7%)
65+ years	348 (0.9%)	222 (1.0%)	570 (0.9%)	17,563 (2%)
*Gender*^b^				
Female	25,133 (63.9%)	13,737 (63.0%)	38,870 (63.5%)	548,305 (59%)
Male	14,222 (36.1%)	8.078 (37.0%)	22,300 (36.5%)	370,126 (40%)
*Province*^c^				
BC	7714 (19.6%)	3940 (18.1%)	11,654 (19.1%)	215,654 (24.8%)
NL	1116 (2.8%)	492 (2.3%)	1608 (2.6%)	40,314 (4.6%)
ON	30,525 (77.6%)	17,383 (79.7%)	47,908 (78.3%)	614,287 (70.6%)
*Baseline Daily Step Count*^d^	6074 ± 3358	6076 ± 3333	6075 ± 3349	5560 ± 2726^e^
*Pre-Intervention (valid weeks)*	11.16 ± 1.7	10.81 ± 2.0	11.03 ± 1.8	n/a
*Intervention (valid weeks)*	11.47 ± 1.4	10.86 ± 2.1	11.25 ± 1.7	n/a

Note: all tests performed on matching data comparing experimental and control group characteristics

^a^Independent samples t-test – *p* < 0.0001, Cohen’s d = 0.042

^b^Chi squared – chi square = 4.819, *p* = 0.028, Cramer’s V = 0.009

^c^Chi squared – chi square = 43.517 *p* < 0.0001, Cramer’s V = 0.027

^d^Baseline Daily Step Count is the mean value calculated based on each user’s daily step count during the first two-weeks they were using the standard steps program; independent samples t-test – *p* > 0.05, Cohen’s d = 0.000661

^e^Baseline daily step count data unavailable for overall Carrot Rewards population, mean and SD from Mitchell et al. (2020)

### Group differences

Controlling for pre-intervention mean daily step counts, ANCOVA showed a significant difference in intervention mean daily step count (F(1, 61,167) = 1515.97, *p* < 0.0001), favouring the experimental group with a small effect (η_p_^2^ = 0.024; Table 2). An estimated marginal means difference of 537 steps per day favoured the experimental group.

**Table 2:** ANCOVA results adjusting for pre-intervention mean daily step count

	Observed Intervention Mean Daily Step Count	Adjusted Intervention Mean Daily Step Count	SE	95% CI	n
Experimental	7712.77	7517.84	8.21	(7501.75 - 7533.93)	39,355
Control^a^	6629.22	6980.93	11.04	(6959.29 – 7002.57)	21,815

^a^Note: R^2^ = .742, Adj. R^2^ = .742

A pairwise t-test was also performed on the total sample (*n* = 20,530 matched pairs of experimental and control users) to compare change in mean daily step count from pre-intervention to intervention for each group. Mean daily step count increased from pre-intervention to intervention for both experimental (1133.92 steps, 95% CI (1110.34 - 1157.50); *p < 0.0001,* Cohen’s d = 0.658) and control (629.49 steps, 95% CI (609.29–649.68); *p < 0.0001*, Cohen’s d = 0.426) groups (Table 3). The experimental group showed an increase of 504 mean steps per day more than the control group from the pre-intervention to intervention periods.

**Table 3:** Pairwise t-test results comparing pre-intervention and intervention mean daily step count

	df	Mean Difference (Intervention – Pre-intervention)	SD	95% CI Lower	95% CI Upper
Experimental	20,529	1133.92	1723.97	1110.34	1157.50
Control	20,529	629.49	1476.33	609.29	649.68

Sensitivity analyses examining users with complete data sets only (those highly-engaged users with data for all 24 weeks) and users who were matched on a 1:1 experimental to control user ratio showed no difference compared to the main findings (Additional files 5 and 6).

### Dose-response relationship

**Fig. 2:**
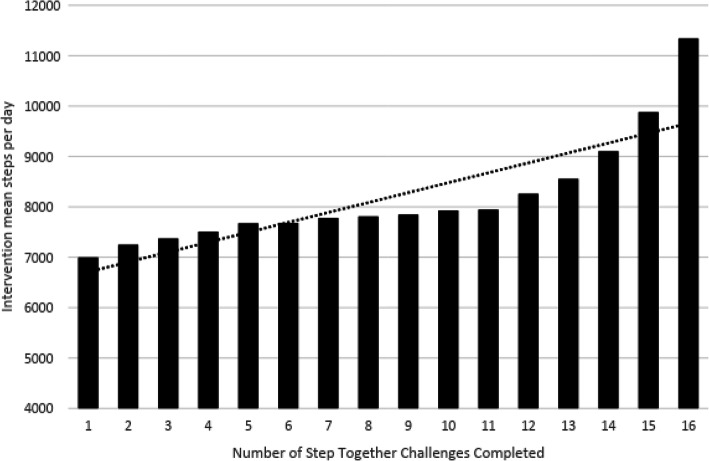
Number of Step Together Challenges completed and corresponding intervention mean daily step count

Linear regression revealed a significant dose-response relationship between the number of STCs completed and mean steps per day [F (1, 14) = 35.834, *p <* 0.0001], with an adjusted *R*^*2*^ of 0.699). On average, participants’ intervention mean daily step count increased 196.80 (unstandardized beta coefficient) for each new STC completed. Descriptive data presented below illustrates this dose-response relationship (Fig. 2 and Additional file 7). Although the increase in intervention mean steps per day appears somewhat exponential when inspecting increases in step count for users completing 15 and 16 STCs in particular, these means actually represent a very small proportion of users (*n* = 270 vs. *n* = 39,355 experimental users in total; see Additional file 7).

## Discussion

### Main findings

In this large quasi-experimental evaluation of the Carrot Rewards app we found that adding team-based incentives increased mean daily step count. Specifically, our experimental group had an increase of 1143 steps whereas the control group saw an increase of 606 steps during the study period – a 537 adjusted mean daily step count difference, or 3759 additional steps per week (roughly equivalent to 40 min of walking). This may be attributed to the application of “herd behaviour”, the behavioural economics principle describing the tendency for individuals to follow others’ behaviours instead of making independent decisions. Carrot Rewards exploited this predictable human tendency by providing real-time feedback on peer progress, as well as by rewarding users only if both achieved at least a few daily goals throughout the Challenge. In doing so, we speculate that STCs may have also served to increase feelings of social connectedness within the small teams, which according to self-determination theory (a global theory of human motivation) promotes quality health behaviour change [53]. A dose-response relationship was also observed with app engagement positively associated with mean daily step count. Each new STC corresponded to about 200 more steps per day.

### Implications

The clinical implications of daily step count increases of these magnitudes are important. For instance, an increase of 1000 steps per day has been associated with significant weight loss in adults and better glycemic control (i.e. lower A1C) in individuals living with type 2 diabetes [15, 54]. Higher step counts in general are also associated with improved mood and overall health ratings, and are inversely related to systolic blood pressure [15, 55]. From a public health perspective, a 1% reduction in the number of Canadians classified as “physically inactive” (fewer than 5000 daily steps) would yield annual healthcare savings of $2.1 billion CAD [56]. With nearly half of the Carrot Rewards users in general accumulating less than 5000 steps per day, a mere 500 to 1000 step increase from baseline values could have broad implications [12]. In fact, a recent 12 month analysis of the Carrot Rewards app suggests approximately 100,000 Canadians moved up from the “physically inactive” category to the “moderately active” (more than 5000 steps/d) [35].

The present study provides further evidence that even very small incentives (STC users earned on average $3.60 CAD over 12 weeks, for a total of about $142,000 CAD) can be implemented as part of a multicomponent intervention to increase PA. Recent evidence suggests that reward size may be less important than other program design features (e.g., incentive timing or form) [57]. It has been suggested that manipulating these other features (nine are outlined by Adams et al. [19] and updated by Mitchell et al. [58]) may help reduce the cost of incentives while maintaining or even boosting effects [21]. In addition to leveraging “herd behaviour”, the very small incentives in this study increased PA because they were offered *immediately* and in the form of *loyalty points*, exploiting two other behavioural economics concepts as well: (a) the human tendency to prefer payoffs close to the present time (“present bias”) [16] and (b) the tendency for people to equate numbers of unclear significance (i.e. the loyalty points used in this case) with greater value (“numerosity effect”) [59]. These and other theoretically-informed manipulations may appeal to governments and insurers looking to deploy PA incentives as efficiently as possible [21].

### Similar studies

Findings from our quasi-experimental examination in a real-world commercial context compliment those from traditional RCT studies examining the impact of team-based incentives on PA or weight loss, in digital and non-digital settings [15, 24–26]. Babcock et al. (2015) found that the number of gym visits was 9–17% higher in the team incentive compared to the individual incentive conditions. In particular, team incentives for teams where members knew each other were more effective than those for anonymous teams [24]. Patel et al. (2016) examined a PA intervention delivered through a research-based smartphone app, combining the social aspects of team incentives with individual-level rewards, much like the incentive scheme used in the present study [26]. In comparing a control condition to individual, team and combined (individual plus team) incentives groups, Patel et al. (2016) found participants in the combined incentives group had a significantly higher mean daily step counts compared to controls (1446 daily step count group difference), whereas the team and individual incentive groups did not outperform controls [26]. Smith-McLallen et al., (2017) also compared a digitally delivered standard walking program to an enhanced program including incentives, feedback and competitive challenges and found that the enhanced group improved their mean daily step count by 726 more than the standard program group over nine months [15]. Finally, a year-long evaluation of the standard Carrot Rewards ‘Steps’ walking program (i.e. before STCs were introduced) found an average increase of 448 and 884 steps per day from baseline for ‘regular’ and ‘committed’ users (i.e. users engaging with the app on 26 to 51 out of 52 weeks, or all 52 weeks, respectively) [12]. It is interesting to note that these pre-post daily step count differences are similar in magnitude to the ones reported here, and that in both studies greater engagement yielded larger effects. This aligns with a growing but still limited (to a small number of RCTs) evidence base suggesting that app exposure is paramount and that greater engagement may produce greater effects [10–12, 33]. Regarding PA incentives in general, caution is warranted given that positive effects are not automatic as seen in RCTs demonstrating the benefits of one incentive design/arm (e.g., chance-based incentives) but not others (e.g., guaranteed incentives) [21].

### Limitations and future directions

This study was not without limitations. First, randomization of participants into experimental and control arms was not logistically feasible within this quasi-experimental design making it difficult to conclude with certainty that the Carrot Rewards STC feature *caused* an increase in PA. For this reason, internal validity may be limited. To increase internal validity in this real-world public health intervention context, we matched experimental participants with similar controls at the study design stage, as well as used three main analytic approaches (i.e. ANCOVA, pairwise t-test, linear regression) to address our primary objective and conducted separate sensitivity analyses (i.e. subgroup ANCOVA and pairwise t-tests) at the analysis stage. Self-selection bias, especially with regard to engagement (i.e. since controls did not engage in a STC during the study period, they may have been less engaged a priori) may have confounded our results despite our best efforts to match experimental users with similar controls. Although we were limited in our ability to match users to a few demographic variables, we were able to match based on baseline daily step count and control for baseline daily step count discrepancies in our analyses. In the future, we suggest other mHealth researchers attempt to maximize group equivalency in quasi-experimental contexts by matching participants using an engagement variable as well (e.g., number of weeks with at least one app open in the past 6 months, number of app features engaged). Unlike what was done here, mHealth researchers should also ensure a 1:1 matching ratio to prevent case-control imbalances. Other design phase strategies to strengthen quasi-experiments, and address selection bias in particular, include interrupted time series designs where multiple observations are evaluated before, during *and* after intervention within the same group [49]. While traditional RCTs strongly prioritize internal validity, this quasi-experimental design seeks to achieve a greater balance between internal and external validity within real-world conditions to facilitate real-world implementation.

Changing seasons (the study started in the typically cold Canadian Winter and ended in the warmer Spring/Summer months) may also have impacted our results. It is reasonable to assume though that this potentially confounding seasonality effect influenced PA step count similarly in both study groups. In addition, smartphone wear time may have differed between study groups. It is possible that PA increases may have been due to the fact that experimental users simply started carrying their smartphones more to get credit for the steps they were taking. As previously cited, it is hard to dis-entangle ‘wear time’ from actual daily step count increases [60]. Device adherence was uniformly high before and during the intervention, however, as seen with similar numbers of valid weeks in the pre-intervention and intervention periods. This was likely optimized with the presence of the individual-level PA incentives, possibly shrinking the gap between measured and actual steps (i.e. assessment error) for both groups [45]. The fact that budget restrictions dictated reward size in this study could also be construed as a limitation, as larger incentives have generally produced larger effects [21]. Lastly, our study did not evaluate the long-term (six or more months ⎯the theoretical threshold of behaviour maintenance) effects of adding team incentives to an existing walking program rewarding users with individual incentives [61]. Future research should evaluate whether combined incentives drive PA improvements over the long-term, and whether adding *unrewarded* team-goals produces similar effects.

## Conclusion

Given the persisting physical inactivity pandemic, there is an urgent need for scalable and effective digital PA interventions [62]. Examinations of real-world effectiveness have been repeatedly called for in the literature highlighting the important contributions made here. We have shown that incorporating concepts from behavioural economics (e.g., herd behaviour) in the design of mHealth app features in a way that leverages *pre-existing* social networks has the potential to improve user engagement and PA behaviours. Specifically, participants using the Carrot Rewards app with team-based incentives accumulated more steps per day during a 12-week intervention period compared to matched controls. The more the STC feature was used, the more participants walked. While the effect of STCs on long-term behaviour change is not clear, their role in increasing feelings of social relatedness (even in a digital environment), and thus the potential for sustained change, would be an interesting line of future inquiry. Taken together, much can be learned from this large-scale evaluation of a top-tier commercial PA app. In particular, more high quality quasi-experimental designs are needed to examine real-world effectiveness in a fast paced mHealth context that does not necessarily lend itself to more carefully controlled RCTs that prioritize internal over external validity.

## Supplementary Information

**Additional file 1.** Detailed description of the matching process.

**Additional file 2.** Study flowchart.

**Additional file 3.** Completed Mobile Application Rating Scale (MARS).

**Additional file 4.** Completed App Behavior Change Scale (scale to assess the potential of apps to promote behavior change).

**Additional file 5.** ANCOVA results adjusting for pre-intervention mean daily step count for total sample, users with complete data sets only (sensitivity), and 1:1 matched users only (sensitivity).

**Additional file 6.** Pairwise t-test results for total sample, users with complete data sets only (sensitivity), and 1:1 matched users only (sensitivity).

**Additional file 7.** Mean steps per day in the pre-intervention and intervention periods by number of Step Together Challenges completed.

**Additional file 8.** Completed TIDieR (Template for Intervention Description and Replication) Checklist.

**Additional file 9.** Completed STROBE Statement—checklist of items to be included in reports of observational studies.

## Data Availability

The datasets used and/or analysed during the current study are available from the corresponding author on reasonable request.
