# Supplementary material for: Adding team-based financial incentives to the Carrot Rewards physical activity app increases daily step count on a population scale: a 24-week matched case control study
Source: Int J Behav Nutr Phys Act. 2020 Nov 19;17:139. doi: 10.1186/s12966-020-01043-1 (PMC7677847; doi:10.1186/s12966-020-01043-1)
Supplement: Supplementary file 1 — Additional file 1. Detailed description of the matching process. [file 12966_2020_1043_MOESM1_ESM.docx]

**Additional file 1:** Detailed description of the matching process.

A maximum ratio of 1:18 control users to experimental users was implemented as this was equivalent to excluding 10% of the study population with the highest matching ratio (the highest ratio prior to exclusion was around 1:250 control users to experimental users. Of the users that were excluded due to large matching ratio, 18 of the experimental users were randomly selected and kept for study analysis with the corresponding matched control user. This was done to avoid excluding users with the most common demographics (i.e., 25 year old female in ON with a baseline step count of 2,500 steps per day) as many experimental users would have these same characteristics therefore would be matched to the same control (e.g., one control user matched with 129 experimental users who have the same matching criteria characteristics). This ensured all demographic characteristics were represented in the analyses.


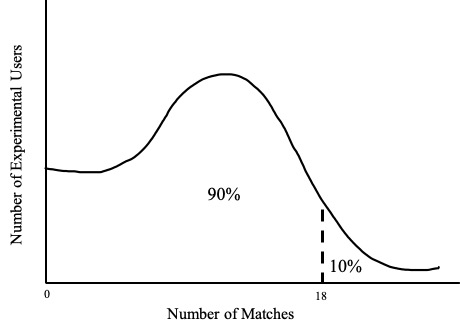


Figure: Explanation of 10% exclusion criteria (10% of participants); maximum ratio of 1:18 control users to experimental users.
