# Supplementary figures and images for: Adding team-based financial incentives to the Carrot Rewards physical activity app increases daily step count on a population scale: a 24-week matched case control study
Source: Int J Behav Nutr Phys Act. 2020 Nov 19;17:139. doi: 10.1186/s12966-020-01043-1 (PMC7677847; doi:10.1186/s12966-020-01043-1)

# **Additional file 2:** Study flowchart.

**
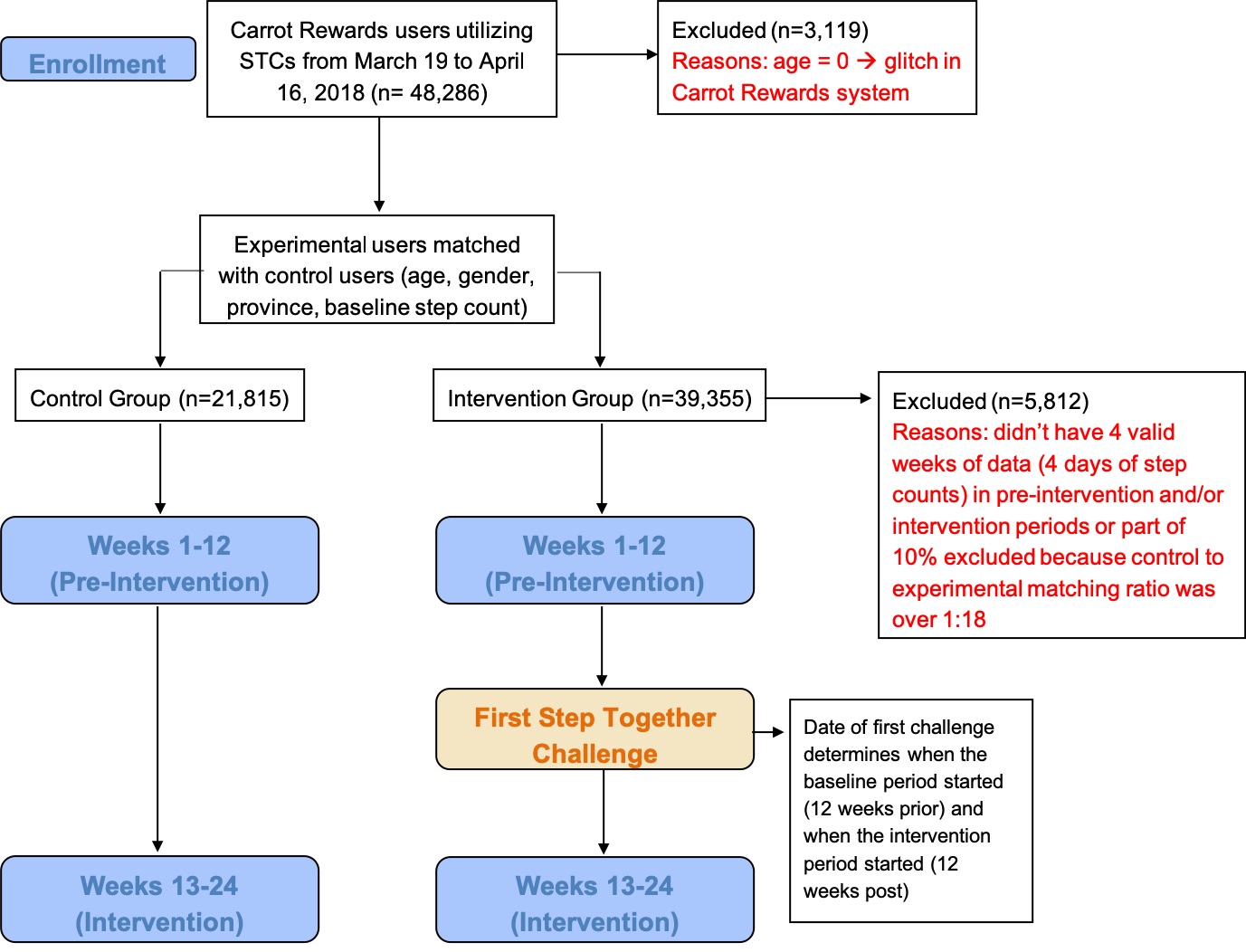
**

Supplement: Supplementary file 2 — Additional file 2. Study flowchart. [file 12966_2020_1043_MOESM2_ESM.docx]
