# Supplementary material for: Adding team-based financial incentives to the Carrot Rewards physical activity app increases daily step count on a population scale: a 24-week matched case control study
Source: Int J Behav Nutr Phys Act. 2020 Nov 19;17:139. doi: 10.1186/s12966-020-01043-1 (PMC7677847; doi:10.1186/s12966-020-01043-1)
Supplement: Supplementary file 5 — Additional file 5. ANCOVA results adjusting for pre-intervention mean daily step count for total sample, users with complete data sets only (sensitivity), and 1:1 matched users only (sensitivity). [file 12966_2020_1043_MOESM5_ESM.docx]

**Additional file 5:** ANCOVA results adjusting for pre-intervention mean daily step count for total sample, users with complete data sets only (sensitivity), and 1:1 matched users only (sensitivity).

| **Category** | **Observed Intervention Mean Daily Step Count** | **Adjusted Intervention Mean Daily Step Count** | **SD** | **n** |
| --- | --- | --- | --- | --- |
| *Total Sample^a^* |  |  |  |  |
| Experimental | 7,712.77 | 7,517.84 | 3,249.04 | 39,355 |
| Control | 6,629.22 | 6,980.93 | 2,984.79 | 21,815 |
| *Complete Data Sets^b^* |  |  |  |  |
| Experimental | 8,014.91 | 7,872.47 | 3,222.09 | 24,413 |
| Control | 7,031.41 | 7,350.86 | 2,834.67 | 10,905 |
| *1:1 Matching Ratio^c^* |  |  |  |  |
| Experimental | 9,025.71 | 8,829.45 | 3,914.19 | 7,090 |
| Control | 7,822.37 | 8,186.15 | 3,716.79 | 3,825 |

^a^Note: R^2^ = .742, Adj. R^2^ = .742

^b^Note: R^2^ = .744, Adj. R^2^ = .744

^c^Note: R^2^ = .777, Adj. R^2^ = .777
