# Supplementary material for: Adding team-based financial incentives to the Carrot Rewards physical activity app increases daily step count on a population scale: a 24-week matched case control study
Source: Int J Behav Nutr Phys Act. 2020 Nov 19;17:139. doi: 10.1186/s12966-020-01043-1 (PMC7677847; doi:10.1186/s12966-020-01043-1)
Supplement: Supplementary file 6 — Additional file 6. Pairwise t-test results for total sample, users with complete data sets only (sensitivity), and 1:1 matched users only (sensitivity). [file 12966_2020_1043_MOESM6_ESM.docx]

**Additional file 6:** Pairwise t-test results for total sample, users with complete data sets only (sensitivity), and 1:1 matched users only (sensitivity).

| **Category** | **df** | **Mean Difference (Intervention – Pre-intervention)** | **Standard Deviation** | **95% CI**  **Lower** | **95% CI**  **Upper** | **P Value** | **Cohen’s d** |
| --- | --- | --- | --- | --- | --- | --- | --- |
| *Total Sample* |  |  |  |  |  |  |  |
| Experimental | 20,529 | 1,133.92 | 1,723.97 | 1,110.34 | 1,157.50 | 0.000 | 0.658 |
| Control | 20,529 | 629.49 | 1,476.33 | 609.29 | 649.68 | 0.000 | 0.426 |
| *Complete Data Sets* |  |  |  |  |  |  |  |
| Experimental | 6,217 | 1,205.05 | 1,703.31 | 1,162.71 | 1,247.40 | 0.000 | 0.708 |
| Control | 6,217 | 703.25 | 1,356.82 | 669.52 | 736.98 | 0.000 | 0.518 |
| *1:1 Matching Ratio* |  |  |  |  |  |  |  |
| Experimental | 3,574 | 1,279.70 | 1,918.09 | 1,216.81 | 1,342.60 | 0.000 | 0.667 |
| Control | 3,574 | 686.81 | 1,718.95 | 630.44 | 743.18 | 0.000 | 0.400 |
|  |  |  |  |  |  |  |  |
