# Supplementary material for: Adding team-based financial incentives to the Carrot Rewards physical activity app increases daily step count on a population scale: a 24-week matched case control study
Source: Int J Behav Nutr Phys Act. 2020 Nov 19;17:139. doi: 10.1186/s12966-020-01043-1 (PMC7677847; doi:10.1186/s12966-020-01043-1)
Supplement: Supplementary file 7 — Additional file 7. Mean steps per day in the pre-intervention and intervention periods by number of Step Together Challenges completed. [file 12966_2020_1043_MOESM7_ESM.docx]

**Additional file 7:** Mean steps per day in the pre-intervention and intervention periods by number of STCs completed.

|  |  | Mean Steps Per Day | |
| --- | --- | --- | --- |
| Number of STCs Completed | n | Pre-Intervention | Intervention |
| 1 | 4,082 | 6,570.27 | 6,997.78 |
| 2 | 3,544 | 6,664.81 | 7,249.37 |
| 3 | 3,048 | 6,721.74 | 7,371.42 |
| 4 | 2,734 | 6,698.75 | 7,509.78 |
| 5 | 2,653 | 6,745.20 | 7,676.61 |
| 6 | 2,764 | 6,694.34 | 7,683.23 |
| 7 | 2,811 | 6,635.96 | 7,779.18 |
| 8 | 2,940 | 6,617.87 | 7,811.53 |
| 9 | 3,156 | 6,569.32 | 7,846.70 |
| 10 | 3,474 | 6,563.46 | 7,924.50 |
| 11 | 3,766 | 6,422.03 | 7,946.07 |
| 12 | 2,560 | 6,295.88 | 8,262.83 |
| 13 | 1,064 | 6,060.79 | 8,556.14 |
| 14 | 442 | 6,199.86 | 9,106.94 |
| 15 | 170 | 6,590.77 | 9,882.42 |
| 16 | 100 | 7,532.08 | 11,344.48 |
