# Supplementary material for: Adding team-based financial incentives to the Carrot Rewards physical activity app increases daily step count on a population scale: a 24-week matched case control study
Source: Int J Behav Nutr Phys Act. 2020 Nov 19;17:139. doi: 10.1186/s12966-020-01043-1 (PMC7677847; doi:10.1186/s12966-020-01043-1)
Supplement: Supplementary file 9 — Additional file 9. Completed STROBE Statement—checklist of items to be included in reports of observational studies. [file 12966_2020_1043_MOESM9_ESM.docx]

STROBE Statement—checklist of items that should be included in reports of observational studies

|  | Item No. | Recommendation | Page  No. | Relevant text from manuscript |
| --- | --- | --- | --- | --- |
| **Title and abstract** | 1 | (*a*) Indicate the study’s design with a commonly used term in the title or the abstract | 1 | 24-week quasi-experimental study |
|  |  | (*b*) Provide in the abstract an informative and balanced summary of what was done and what was found | 2 | Participants who used Carrot Rewards and STCs (experimental group) were matched with those who used Carrot Rewards only (controls) on age, gender, province and baseline mean daily step count (±500 steps).  Adding team-based incentives to the Carrot Rewards app increased mean daily step count. A dose-response relationship was observed with app engagement (expressed in number of STCs completed) positively associated with mean daily step count. |
| Introduction | | | |  |
| Background/rationale | 2 | Explain the scientific background and rationale for the investigation being reported | 3 | mHealth apps have potential to reach large populations at relatively low cost. Their effectiveness, however, is often limited by low user engagement with 90% of mHealth apps being deleted within 30 days. |
| Objectives | 3 | State specific objectives, including any prespecified hypotheses | 5+6 | Examine the impact of adding team incentives to the Carrot Rewards app already rewarding users for individual-level daily step goal completions on mean daily step count. A secondary objective was to determine whether a dose-response relationship existed between the number of STCs completed (an app engagement measure) and mean daily step count. |
| Methods | | | |  |
| Study design | 4 | Present key elements of study design early in the paper | 6 | A 24-week retrospective pre-post matched pairs (quasi-experimental) design. Control participants were matched with existing experimental participants on age, gender, province and baseline step count (±500 steps). The pre-intervention period was defined as the 12 weeks preceding experimental users’ first STC (Study Weeks 1-12). The intervention period included the 12 weeks following the initiation of the first STC (Study Weeks 13-24). |
| Setting | 5 | Describe the setting, locations, and relevant dates, including periods of recruitment, exposure, follow-up, and data collection | 6 | Three provinces the app was launched (i.e. British Columbia (BC), Newfoundland and Labrador (NL), Ontario (ON)). The experimental group included participants using the STC feature for the first time between March 19 and April 16, 2018. The pre-intervention period was defined as the 12 weeks preceding experimental users’ first STC (Study Weeks 1-12). The intervention period included the 12 weeks following the initiation of the first STC (Study Weeks 13-24). |
| Participants | 6 | (*a*) *Cohort study*—Give the eligibility criteria, and the sources and methods of selection of participants. Describe methods of follow-up  *Case-control study*—Give the eligibility criteria, and the sources and methods of case ascertainment and control selection. Give the rationale for the choice of cases and controls  *Cross-sectional study*—Give the eligibility criteria, and the sources and methods of selection of participants | 6 | The experimental group included participants using the STC feature for the first time between March 19 and April 16, 2018 (the first month STC was available). Control participants were drawn from the cohort of Carrot Rewards users who had enabled the ‘Steps’ walking program but had not engaged in a STC during the study period. Participants were required to have valid pre-intervention and intervention study periods, consisting of a minimum of four weeks of daily step count data in each period—a valid week was operationally defined as a minimum of four days with step counts between 1,000 and 40,000 inclusive |
|  |  | (*b*) *Cohort study*—For matched studies, give matching criteria and number of exposed and unexposed  *Case-control study*—For matched studies, give matching criteria and the number of controls per case | 6 | Control participants were matched with existing experimental participants on age, gender, province and baseline step count (±500 steps). |
| Variables | 7 | Clearly define all outcomes, exposures, predictors, potential confounders, and effect modifiers. Give diagnostic criteria, if applicable | 8 | The primary outcome was weekly mean daily step count. Number of STCs completed. |
| Data sources/ measurement | 8* | For each variable of interest, give sources of data and details of methods of assessment (measurement). Describe comparability of assessment methods if there is more than one group | 8 | Weekly mean daily step count as measured by built-in smartphone accelerometers or Fitbit trackers. Number of STCs completed was defined as any STC that was started and finished, irrespective of whether the challenge was completed successfully or not. |
| Bias | 9 | Describe any efforts to address potential sources of bias | 8 | primary outcome was weekly mean daily step count as measured by built-in smartphone accelerometers or Fitbit trackers. In recent validation studies, the iPhone step counting feature, as well as those for Android smartphones and Fitbit trackers were accurate in laboratory and field conditions.36-39 |
| Study size | 10 | Explain how the study size was arrived at | 6 | Participants using the STC feature for the first time between March 19 and April 16, 2018 (the first month STC was available) matched with controls. |

Continued on next page

| Quantitative variables | 11 | Explain how quantitative variables were handled in the analyses. If applicable, describe which groupings were chosen and why | 8 | Primary outcome was weekly mean daily step count as measured by built-in smartphone accelerometers or Fitbit trackers.  Self-reported demographics (i.e. age, gender, province) and number of STCs completed were also collected. Number of STCs completed was defined as any STC that was started and finished, irrespective of whether the challenge was completed successfully or not. |
| --- | --- | --- | --- | --- |
| Statistical methods | 12 | (*a*) Describe all statistical methods, including those used to control for confounding | 9 | Chi-square and independent t-tests were conducted to examine group equivalency on demographic measures. Controlling for pre-intervention mean daily step count, ANCOVA was performed to examine group differences in intervention period mean daily step count. |
|  |  | (*b*) Describe any methods used to examine subgroups and interactions | 9 | A pairwise t-test was also used to examine the mean daily step count change over time (pre-intervention vs. intervention) for each group.  Linear regression was performed to determine the relationship between the number of STCs completed and intervention period mean daily step count. |
|  |  | (*c*) Explain how missing data were addressed | 6 | Participants were required to have valid pre-intervention and intervention study periods, consisting of a minimum of four weeks of daily step count data in each period—a valid week was operationally defined as a minimum of four days with step counts between 1,000 and 40,000 inclusive. (Any data not meeting this criteria was excluded.) |
|  |  | (*d*) *Cohort study*—If applicable, explain how loss to follow-up was addressed  *Case-control study*—If applicable, explain how matching of cases and controls was addressed  *Cross-sectional study*—If applicable, describe analytical methods taking account of sampling strategy | 6 | Control participants were matched with existing experimental participants on age, gender, province and baseline step count (±500 steps). |
|  |  | (*e*) Describe any sensitivity analyses | 9 | Sensitivity analyses were performed on users with complete data sets only, and users with a 1:1 control to experimental participant matching ratio (Appendix Tables 1-6). |
| Results | | | | |
| Participants | 13* | (a) Report numbers of individuals at each stage of study—eg numbers potentially eligible, examined for eligibility, confirmed eligible, included in the study, completing follow-up, and analysed | 10 | Study Population  (n=61,170), Experimental  (n=39,355), Control  (n=21,815) |
|  |  | (b) Give reasons for non-participation at each stage |  | N/A |
|  |  | (c) Consider use of a flow diagram |  | N/A |
| Descriptive data | 14* | (a) Give characteristics of study participants (eg demographic, clinical, social) and information on exposures and potential confounders | 9 | Carrot Rewards’ study sample characteristics, average baseline mean daily count, and average number of valid weeks in the pre-intervention and intervention period can be found in Table 1. |
|  |  | (b) Indicate number of participants with missing data for each variable of interest |  | N/A |
|  |  | (c) *Cohort study*—Summarise follow-up time (eg, average and total amount) |  |  |
| Outcome data | 15* | *Cohort study*—Report numbers of outcome events or summary measures over time |  |  |
|  |  | *Case-control study—*Report numbers in each exposure category, or summary measures of exposure | 10 | Experimental  (n=39,355), Control  (n=21,815) |
|  |  | *Cross-sectional study—*Report numbers of outcome events or summary measures |  |  |
| Main results | 16 | (*a*) Give unadjusted estimates and, if applicable, confounder-adjusted estimates and their precision (eg, 95% confidence interval). Make clear which confounders were adjusted for and why they were included | 11 | Controlling for pre-intervention mean daily step counts, ANCOVA showed a significant difference in intervention mean daily step count (F(1, 61 167)=1,515.97, p<0.000), favouring the experimental over the control group with a small effect (ηp2=0.024; Table 2). An estimated marginal means difference of 537 steps per day favoured the experimental group. |
|  |  | (*b*) Report category boundaries when continuous variables were categorized | 7 | Step counts between 1,000 and 40,000 inclusive |
|  |  | (*c*) If relevant, consider translating estimates of relative risk into absolute risk for a meaningful time period |  | N/A |

Continued on next page

| Other analyses | 17 | Report other analyses done—eg analyses of subgroups and interactions, and sensitivity analyses | 11+12 | A pairwise t-test showed mean daily step count increased from pre-intervention to intervention for both experimental (1133.92 steps, 95% CI (1,110.34 - 1,157.50); p < 0.000, Cohen’s d = 0.658) and control (629.49 steps, 95% CI (609.29 - 649.68); p < 0.000, Cohen’s d = 0.426) groups (see Appendix 6).  The sensitivity analyses examining users with complete data sets (those with data for all 24 weeks of data) and users who were matched on a 1:1 experimental to control user ratio showed no difference compared to the main findings (Appendix 5 and 6).  Linear regression revealed a significant dose-response relationship between the number of STCs completed and mean steps per day ([F (1, 14) = 35.834, p<0.000], with an adjusted R2 of 0.699). On average, participants’ intervention mean daily step count increased 196.80 (unstandardized beta coefficient) for each new STC completed. |
| --- | --- | --- | --- | --- |
| Discussion | | | | |
| Key results | 18 | Summarise key results with reference to study objectives | 12+13 | Adding team-based incentives to the standard steps program increased mean daily step count.  A dose-response relationship was also observed with app engagement (expressed in number of STCs completed) being positively associated with intervention period mean daily step count. |
| Limitations | 19 | Discuss limitations of the study, taking into account sources of potential bias or imprecision. Discuss both direction and magnitude of any potential bias | 14+15 | The quasi-experimental design, for instance, makes it difficult to conclude with confidence that the Carrot Rewards app caused an increase in PA.  Self-selection bias may have confounded our results despite our best efforts to match experimental users with similar controls.  Changing seasons (the study started in the typically cold Canadian Winter and ended in the warmer Spring/Summer months) may also have impacted our results.  Lastly, our study did not evaluate the long-term (six or more months ⎯ the theoretical threshold of behaviour maintenance) effects of adding team incentives to an existing steps program rewarding users with individual incentives. |
| Interpretation | 20 | Give a cautious overall interpretation of results considering objectives, limitations, multiplicity of analyses, results from similar studies, and other relevant evidence | 16 | Participants using the Carrot Rewards standard steps walking program with team-based incentives accumulated more steps per day during a 12-week intervention period compared to matched controls. |
| Generalisability | 21 | Discuss the generalisability (external validity) of the study results | 14 | Although internal validity was limited, there was strong external validity due to the large sample size and observational nature of the study making the results more generalizable. |
| Other information | |  | | |
| Funding | 22 | Give the source of funding and the role of the funders for the present study and, if applicable, for the original study on which the present article is based | 1 | EP received the Ontario Graduate Scholarship to support this work. |

*Give information separately for cases and controls in case-control studies and, if applicable, for exposed and unexposed groups in cohort and cross-sectional studies.

**Note:** An Explanation and Elaboration article discusses each checklist item and gives methodological background and published examples of transparent reporting. The STROBE checklist is best used in conjunction with this article (freely available on the Web sites of PLoS Medicine at http://www.plosmedicine.org/, Annals of Internal Medicine at http://www.annals.org/, and Epidemiology at http://www.epidem.com/). Information on the STROBE Initiative is available at www.strobe-statement.org.
